# The Advancement of Appendicitis in Children in the Pre-Pandemic and the Pandemic Year

**DOI:** 10.3390/jcm13206137

**Published:** 2024-10-15

**Authors:** Marcin Jerzy Owczarzak, Mateusz Biela, Mateusz Paplicki, Małgorzata Rąpała, Joanna Jakubaszko-Jabłońska, Marzena Kozakiewicz, Piotr Miśkiewicz, Kinga Niewińska, Ewa Joanna Godzińska, Jan Godziński

**Affiliations:** 1 Department of Pediatric Surgery, Marciniak Hospital, 54-049 Wrocław, Poland; mrapala@poczta.onet.pl (M.R.); jan.godzinski@umw.edu.pl (J.G.); 2 Division of Pediatric Traumatology and Emergency Medicine, Faculty of Medicine, Wroclaw Medical University, 50-345 Wrocław, Poland; mateusz.paplicki@umw.edu.pl (M.P.); joanna.jakubaszko-jablonska@umw.edu.pl (J.J.-J.); kozakiewicz.marzena@gmail.com (M.K.); piotr.miskewicz@umw.edu.pl (P.M.); kinga.i.niewinska@gmail.com (K.N.); 3 Department of Pediatric Surgery and Urology, University Hospital, 50-556 Wrocław, Poland; 4 Department of Pediatrics, Endocrinology, Diabetology and Metabolic Diseases, Wroclaw Medical University, 50-368 Wrocław, Poland; mateusz.biela@umw.edu.pl; 5 Laboratory of Ethology, Nencki Institute of Experimental Biology of the Polish Academy of Sciences, 02-093 Warsaw, Poland; e.godzinska@nencki.edu.pl

**Keywords:** acute appendicitis, pediatric, COVID-19, SARS-CoV-2, coronavirus, pandemic, surgery

## Abstract

**Background:** The COVID-19 pandemic affected the health of millions of people, both directly through infection and indirectly through delayed diagnosis and treatment of non-COVID-19 illnesses. The aim of this study was to check the impact of the COVID-19 pandemic on the diagnosis and treatment of appendicitis in children. **Methods**: The study was carried out at the Department of Paediatric Surgery of the Marciniak Hospital (Wrocław, Poland) and covered two periods, the pre-pandemic one (P1, 01/03/2019–29/02/2020) and the pandemic one (P2, 01/03/2020–28/02/2021). **Results**: The number of admissions of patients with suspected appendicitis and observation-only patients decreased during the pandemic (400/289 and 226/160, respectively). Although the number of operated children was similar during both analyzed periods (P1: 174, P2: 160), the rate of surgical interventions was significantly higher during P2 (55.4%) than during P1 (43.5%) (χ^2^ test: *p* = 0.00272). The values of the variables quantifying disease progression and severity of inflammation, selected inflammation-related parameters detected by laboratory blood tests, latencies from the onset of symptoms to the admission and from the admission to the operation, and total duration of hospitalization did not differ significantly between the pre-pandemic and pandemic periods. **Conclusions**: These results show that the COVID-19 pandemic led to more rigorous and careful triage of pediatric patients suspected of acute appendicitis that did not have a negative impact on patient outcomes.

## 1. Introduction

Acute appendicitis, an inflammatory process that affects the diverticulum of the cecum and induces acute abdominal symptoms, is the most common childhood disease that has to be treated by surgical intervention [1]. Research on the epidemiology of appendicitis carried out in the United States demonstrated that the annual incidence of this illness increases from the rate of 1 to 6 per 10,000 children under 4 years of age to 19–28 per 10,000 children younger than 14 years [2,3].

For acute appendicitis, urgent diagnosis and rapid treatment are mandatory, as progressing inflammatory processes may lead to the perforation of the appendix and, consequently, to peritonitis, which may result in sepsis and may even lead to the death of the patient [4,5]. The procedure of choice in the treatment of acute appendicitis consists of the surgical resection of the infection focus, the so-called appendectomy, which may be performed with the use of either the classical method or the laparoscopic one [6]. Non-surgical treatment of acute appendicitis in children may also be applied, but it is not widespread [7]. The prognosis in uncomplicated appendicitis is good, and the mortality rate is relatively low (0.3%), although it increases to 1.7% after perforation [4].

Since the initial report of coronavirus infection in Wuhan, China, on 29 December 2019, the SARS-CoV-2 (COVID-19) pandemic attained global distribution and strongly influenced our habits and lifestyles on a global scale [8]. Necessary precautions, lock-downs, and the recommendations to avoid crowded areas were applied to decrease the risk of the virus spread. That strategy of the prevention of disease spread was also applied in the context of contacts with the medical care providers and patients visiting medical centers. During the pandemic, hospitals primarily focused on treating COVID-19 patients, while other medical services were significantly reduced or nearly halted. Non-COVID-19 patients were admitted only when strictly necessary. This situation was expected to negatively impact the treatment of non-COVID-19 diseases due to delays in diagnosis and care. These delays could lead to disease progression and worse outcomes due to more advanced stages of untreated conditions. However, fewer hospital admissions occurred for cases based on weaker clinical suspicions. Logically, that decrease should not have had negative consequences for the outcomes of the patients, as it resulted from more rigorous but still very careful triage of pediatric patients suspected of acute appendicitis. In addition, during the first few weeks of the pandemic, the advantage of open operations over laparoscopy was suggested, as it was believed that laparoscopy might act as an aerosol-generating procedure that might contribute to the facilitation of virus transmission and COVID-19 spread [9]. Moreover, we also know that COVID-19 may induce appendicitis in both children and adults [10,11]. On the other hand, there are reports suggesting that acute appendicitis may also be a rare side effect of COVID-19 vaccination, particularly in younger populations [12]. In the case of all medical conditions including appendicitis, non-operative management (including observation, dietary restrictions and antibiotic therapy), was also recommended to avoid surgery, if deemed to represent an acceptable alternative treatment option [9,13].

The main aim of this study was to examine the impact of the COVID-19 pandemic on pediatric patients with acute appendicitis. For that purpose, we examined the number of hospitalized patients and the rates of those who underwent surgery. We also analyzed the severity of appendicitis in our patients and inflammation-related parameters identified through laboratory blood tests. Additionally, we assessed the time from the onset of appendicitis symptoms to hospital admission, the time from admission to surgery, and the total duration of hospitalization. According to our main hypothesis, the outbreak of the COVID-19 pandemic should have resulted in delayed diagnoses and treatment and, as a consequence, in worse outcomes for children suffering from acute appendicitis.

## 2. Materials and Methods

### 2.1. Study Design

The data analyzed in this study were collected during two periods, each consisting of one year. The first study period (the pre-pandemic period, P1; 1 March 2019–29 February 2020) took place immediately prior to the outbreak of the COVID-19 pandemic in Poland, and the second one (the pandemic period, P2; 1 March 2020–28 February 2021) took place immediately afterward, during the first year of that pandemic. These two periods were retrospectively analyzed. We compared the numbers of patients admitted to the hospital with the suspicion of acute appendicitis, the latencies from the onset of symptoms of appendicitis to the admission to the hospital, the operability rate, the degree of advancement of appendicitis, basic inflammation-related parameters detected by laboratory blood tests, the latencies from the admission to the hospital to the operation, and the total duration of the hospitalization. The list of categories used in the evaluation of the degree of advancement of appendicitis is provided at the start of Section 3.4, “Degree of advancement of the disease”. Full information about basic inflammation-related parameters detected by laboratory blood tests and the results of their analysis is provided in Section 3.5, “Results of Laboratory Blood Tests”. All patients analyzed in the present study were younger than 18 years of age and were admitted to the Department of Paediatric Surgery, Marciniak Hospital, Wrocław, Poland, during the period P1 or P2.

The diagnosis of acute appendicitis was made on the basis of anamnesis, physical examination, laboratory tests investigating blood parameters related to an inflammatory state, ultrasonography, and histopathological examination after appendectomy. 

### 2.2. Statistical Methods

For each of the investigated variables, the following parameters were calculated: mean (X), standard deviation (SD), range (minimum, maximum), lower and upper quartile (25Q, 75Q), and median (M). The statistical significance of the differences between the compared groups was calculated by one-way analysis of variance (ANOVA). Alternatively, if the variances in the analyzed groups were not homogeneous, the non-parametric Mann–Whitney U test was used. The homogeneity of variance was examined by the Levene’s test. In the case of the rates of occurrence, the statistical significance of differences between the compared groups was checked by means of chi-square tests with the corresponding numbers of the degrees of freedom (χ^2^df). In all statistical tests, a *p*-value of less than 0.05 was required to reject the null hypothesis. Statistical analysis was performed using the EPIINFO Ver. 7.2.3.1 and Statistica Ver. 13.3. software packages.

## 3. Results

### 3.1. Patients: Gender, Age, and Symptoms Reported on Admission

The principal indices characterizing the pediatric patients in this study were similar in both analyzed groups. These groups included children treated during P1 and P2. The female-to-male ratio did not differ significantly between the two analyzed periods (χ^2^ = 0.295, *p* = 0.587). Females constituted 43.3% of the patients treated during P1 (173 out of 400) and 41.2% of the patients treated during P2 (119 out of 289).

The age of the patients varied from 0 to 17.9 years, with an average age of 10.8 years in the group treated during P1 and 10.9 years in P2. The median of that variable was equal to 11.0 in the case of both analyzed periods. Like gender, the age of the patients also did not show significant differences between the compared periods, which were compared by the Mann–Whitney U test.

On admission, all patients in both groups presented complaints of abdominal pain. Most often, patients reported pain in the location typical for acute appendicitis, i.e., in the right lower quadrant of the abdomen. Predominantly, the pain lasted up to 12 h in both compared groups (P1 33.9% vs. P2 31.3%; χ^2^ = 0.457, *p* = 0.499).

Vomiting was another common symptom, reported by 110 patients (27.5%) in P1 and 91 patients (31.5%) in P2. The rate of the patients with reported vomiting also did not differ significantly between the compared periods (χ^2^ = 1.11, *p* = 0.293).

### 3.2. Admissions to the Hospital

**Figure 1:**
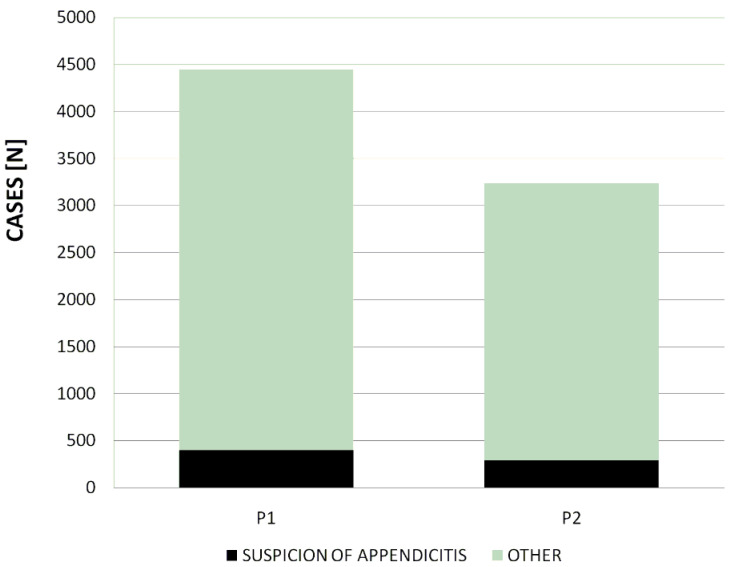
Number of patients hospitalized during the pre-pandemic period (P1) and the pandemic period (P2).

The total number of admissions to the Department of Paediatric Surgery of the Marciniak Hospital in Wrocław (Poland) was lower during the pandemic year (P2: 3235 admissions) than during the pre-pandemic one (P1: 4443 admissions). The decrease in the total number of admissions that took place during the first year of the COVID-19 pandemic was thus relatively important (27.2%, see also Figure 1). 

The number of patients admitted on the basis of the suspicion of appendicitis was also lower during the pandemic period (289/3235, 8.9%) than during the preceding pre-pandemic one (400/4443; 9.0%) (Figure 1). A relatively important (27.8%) decrease in the total number of admissions related to the suspicion of appendicitis was thus observed during the first year of the pandemic. The ratio of the patients admitted on the basis of that suspicion to those admitted because of other medical problems did not differ between the two compared periods (χ^2^ = 0.004, *p* = 0.948).

Latencies from the onset of the initial symptoms suggesting appendicitis to the admission of the patient to the Emergency Department of the Marciniak Hospital showed no significant differences between the two compared periods.

### 3.3. Operability

**Figure 2:**
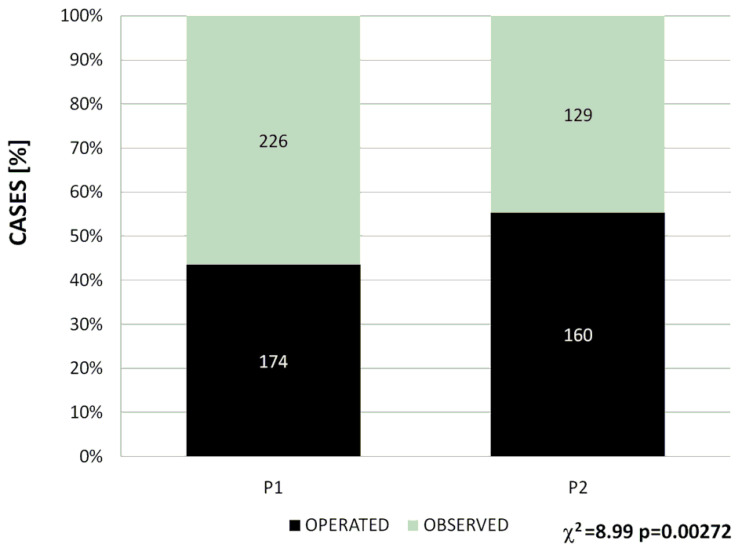
The difference in the operability rates between the pre-pandemic period (P1) and the pandemic period (P2).

As already stated, although the numbers of patients admitted for appendicitis during each of the two compared periods were different, these patients constituted the same proportion of the whole group of patients admitted during these periods (9.0% vs. 8.9% during P1 and P2, respectively). However, the patients with the suspicion of appendicitis admitted to the hospital during the compared periods differed with respect to their further treatment. The number of children submitted solely for observation and then dismissed after the exclusion of appendicitis was significantly higher during P1 (226/400; 56.5%) than during P2 (129/289; 44.6%) (χ^2^ test: *p* = 0.002). In other words, during the first year of the COVID-19 pandemic in Poland, there occurred a 21.1% decrease in the rate of pediatric patients with the suspicion of appendicitis who were submitted only to observation during their hospitalization and then dismissed without having been operated on. The numbers of operated cases were similar during both compared periods: 174 patients during P1 and 160 patients during P2, with a relatively slight (8.0%) decrease during the pandemic period. However, the operability rate was significantly higher during P2 (55.4%) than during P1 (43.5%) (Figure 2).

### 3.4. Degree of Advancement of the Disease

**Figure 3:**
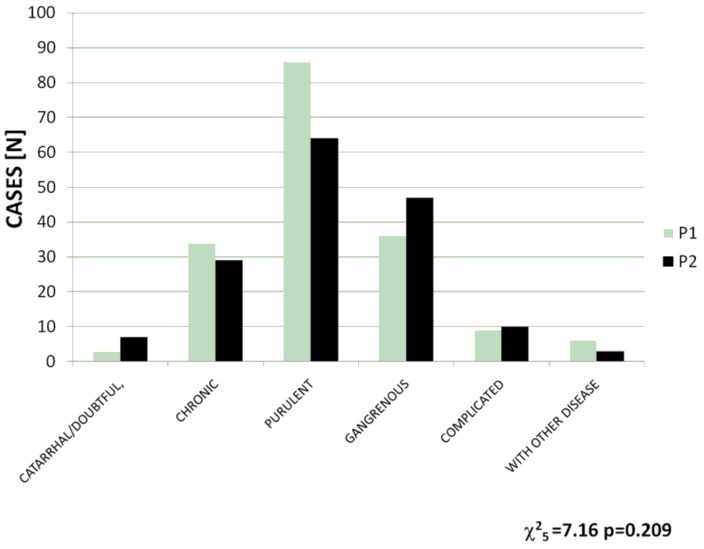
Incidence of particular forms of appendicitis in the pre-pandemic period (P1) and the pandemic period (P2).

Another factor analyzed in the study was the degree of advancement of the disease observed in the operated children. To compare the findings obtained during two analyzed periods, histopathological grading of appendicitis was used, and appendicitis was classified as belonging to one of the following six categories: catarrhal or doubtful, chronic, purulent, gangrenous, complicated (e.g., a peri-appendicular abscess), or associated with other diseases. The following numbers of cases and rates of the specific forms of appendicitis were recorded during the two compared periods: catarrhal or doubtful: 3 (1.7%) vs. 7 (4.4%); chronic: 34 (19.5%) vs. 29 (18.1%); purulent: 86 (49.4%) vs. 64 (40%); gangrenous: 36 (20.6%) vs. 47 (29.3%); complicated: 9 (5.1%) vs. 10 (6.2%); associated with other diseases: 6 (3.4%) vs. 3 (1.8%). No significant differences between the compared periods were discovered (χ^2^ = 7.16, *p* = 0.209) (see also Figure 3).

### 3.5. Results of Laboratory Blood Tests

**Figure 4:**
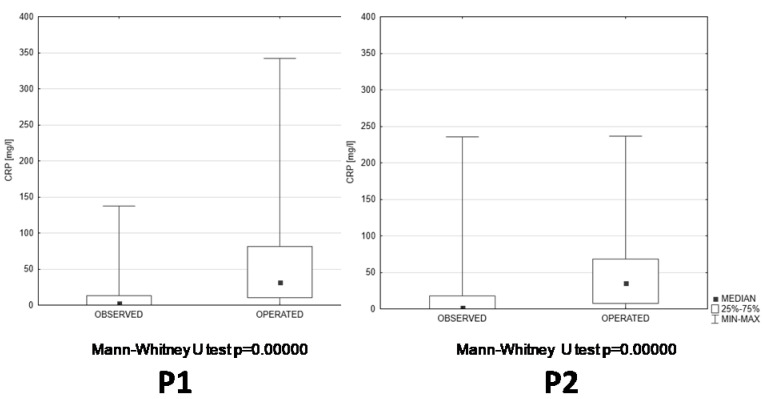
Comparison of CRP in two subcategories of patients (observed and operated) hospitalized during P1 and P2; CRP—C-reactive protein [mg/L]; P1—pre-pandemic period; P2—pandemic period.

**Figure 5:**
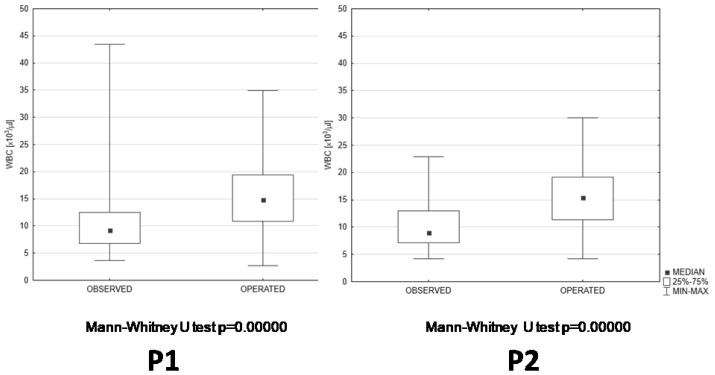
WBC comparison in two subcategories of patients (observed and operated) hospitalized during P1 and P2; WBC—white blood cell [10^3^/µL]; P1—pre-pandemic period; P2—pandemic period.

The results of basic laboratory blood tests checking the advancement of inflammatory reactions revealed that the C-reactive protein concentrations (Figure 4), white blood cell counts (Figure 5), and lymphocyte and neutrophil counts (Table 1) were at similar levels during both compared periods. However, C-reactive protein concentrations and white blood cell counts showed highly significant differences between the patients that were only observed and dismissed without surgical treatment and the patients that had to undergo surgical interventions (Figure 4 and Figure 5). These differences between observation-only and operated patients were observed during both compared periods (Figure 4 and Figure 5).

### 3.6. Latency from the Admission to the Hospital to the Operation and Total Duration of Hospitalization

The analysis of the latencies from the admission to the hospital to the operation (LAOs) did not reveal significant differences between the compared periods (Table 1).

The total duration of the hospitalization (TDH) tended to be shorter during the pandemic period than during the preceding pre-pandemic period (Table 1). However, that trend was not significant (*p* = 0.0774).

**Table 1:** Patients submitted to appendectomy—selected results.

Results	Period P1 (Pre-Pandemic) 1 March 2019–29 February 2020								Period P2 (Pandemic) 1 March 2020–28 February 2021								*p*
	N	X	SD	Min	25Q	M	75Q	Max	N	X	SD	Min	25Q	M	75Q	Max	
CRP [mg/L]	138	65.4	8.8	0.1	10	31.6	81.6	342	136	47.7	50.9	0	8	35.6	68.1	237	0.225 *
WBCs [10^3^/µL]	140	15.5	6.2	2.7	10.9	14.9	19.4	34.9	137	15.5	5.6	4.2	11.4	15.4	19.1	30.0	0.910 **
LYMPH [10^3^/µL]	140	2.08	1.94	0.42	1.16	1.79	2.39	17.4	137	1.84	0.96	0.33	1.14	1.67	2.42	6.05	0.189 **
NEUTR [10^3^/µL]	140	12.7	7.9	2	8.3	11.7	16.3	74.6	137	12.3	5.3	1.9	8.7	11.9	15.4	26.8	0.571 **
LAO (h)	167	0.305	0.709	0	0	0	0	6	156	0.269	0.615	0	0	0	0	4	0.626 **
TDH [days]	174	8.01	2.64	4	7	7	8	24.0	160	7.44	3.27	2	6	7	8	29.0	0.0774 **

CRP—C-reactive protein; WBCs—white blood cells; LYMPH—lymphocytes; NEUTR—neutrophils; LAO—latency from the admission to the hospital to surgery; TDH—total duration of hospitalization; N—number of cases; X—mean; SD—standard deviation; min—minimum; 25Q—lower quartile; M—median; 75Q—upper quartile; max—maximum; * Mann–Whitney U test; ** one-way ANOVA

## 4. Discussion

### 4.1. Main Aims, Outcomes, and Implications of Our Research

Our retrospective research has been carried out to evaluate the impact of the COVID-19 pandemic on pediatric patients with acute appendicitis by comparing a wide range of data obtained during the pre-pandemic one-year period (1 March 2019–29 February 2020) and the subsequent initial year of the pandemic (1 March 2020–28 February 2021). Similarly to some other (but not all) studies devoted to this question, our study did not reveal major negative outcomes either of the pandemic itself or of modified regulations of hospital admissions implemented in response to its outbreak. More rigorous criteria adopted during the pandemic periods even seemed to help to reduce unnecessary hospital admissions. This finding is thus of importance for everyday clinical practice, as it indicates that it may be possible to prevent over-admission and optimize hospital resources without negative outcomes for the patients.

### 4.2. Factors Responsible for Delays in Starting the Treatment of Appendicitis during COVID-19 Pandemic

The delay in starting adequate treatment logically implies the progression of the majority of diseases during the waiting period. Intuitively, such a delay should result in longer hospitalization, more intensive treatments, a higher frequency of complications, and poorer outcomes. Appendicitis is characterized by stable population occurrence, a well-known natural history, and quasi-linear progression from a mild catarrhal form to purulent and gangrenous inflammation, often associated with peritonitis. This natural history of the progression of appendicitis with its quasi-linear characteristics makes this disease particularly suitable to be used as a model illness that may contribute in a substantial way to broadening our knowledge about diseases characterized by similar progression dynamics. We also should bear in mind that abdominal pain appearing in childhood is nearly always evaluated in the optics of appendicitis, leading to hospital admissions for observation, even if many patients are later discharged without a diagnosis of appendicitis.

Our study analyzed the impact of the COVID-19 pandemic on the severity of acute appendicitis in pediatric patients at the Marciniak Hospital in Wrocław, Poland. All pediatric patients requiring surgical intervention are treated in our Department of Paediatric Surgery, and those suspected of needing surgery are admitted for observation. A typical diagnostic path begins in the Emergency Department with anamnesis, physical examination, abdominal ultrasonography, and laboratory blood tests. Various factors that may delay the reporting of a child suspected of having acute appendicitis to the Emergency Department include insufficient level of knowledge about the symptoms of appendicitis possessed by the child’s caregivers, symptom severity, misdiagnosis, and socioeconomic factors [14,15]. The COVID-19 pandemic introduced additional sources of delay due to fear of infection and hospital overcrowding.

The factors that might affect the start of diagnosis and treatment of pediatric patients with acute appendicitis during the COVID-19 pandemic and, therefore, exert an impact on the advancement of the disease were also analyzed by Snapiri et al. [16]. According to that study, carried out in Israel, the predominant reason for these delays was parents’ reluctance to bring children with mild, short-lasting symptoms to hospitals, fearing potential COVID-19 exposure. These delays resulted, however, in a higher rate of post-operative complications [16]. In a similar analysis of these factors, Pantalos et al. also named changes to public health measures, increased use of telemedicine, COVID-19 infection with concurrent acute appendicitis, recurrence of appendicitis after non-operative management, and increased time to intraoperative diagnosis [17]. Among other potential reasons for delays, we can also point out the local guidelines introduced in Poland during the COVID-19 pandemic. Each patient newly admitted to the hospital had to be tested for SARS-CoV-2 infection and isolated from other patients until the result of the test became known and proved to be negative, or isolated permanently if the result of that test proved to be positive. The indications for surgery were strictly followed. Furthermore, the availability of operating rooms decreased due to new sanitary guidelines including special decontamination procedures, as well as due to the fact that part of the operating rooms started to be dedicated exclusively to COVID-19 patients. The risk of SARS-CoV-2 transmission from patients to medical staff was also taken into account. This included the use of personal protective equipment by the staff and enhanced infection control protocols in operating rooms.

### 4.3. The Effects of the COVID-19 Pandemic on Severity and Outcomes of Appendicitis in Pediatric Patients

Our initial hypothesis was that patients admitted during the pandemic would present more advanced stages of appendicitis due to treatment delays. Data supporting such a hypothesis were recently reported by Gerall et al., who observed an increased severity of symptoms, higher perforation rates, more frequent complications, and longer recovery times in pediatric patients with appendicitis during the pandemic in New York [18]. Similarly, in Poland, Pawelczyk et al. reported higher complication rates, more frequent drainages, increased conversion from laparoscopic to open surgeries, and longer hospitalizations for appendectomies carried out during the pandemic period [19]. Increased rates of pediatric perforated appendicitis observed during the COVID-19 pandemic and related to stay-at-home ordinances and delayed hospital admission taking place during the COVID-19 pandemic were also reported by Esparaz et al. in Great Britain, Li and Saleh in the USA, and Ergün et al. in Turkey [20,21,22]. They were also reported by Pogorelić et al. and, most recently, by Miscia et al. in review papers analyzing numerous cases of pediatric appendicitis recorded during the COVID-19 pandemic and the pre-pandemic period [23,24]. A similar conclusion (a higher rate of complicated appendicitis observed in adults during the COVID-19 pandemic period) was also presented by Orthopoulos et al. in a study devoted to the effect of the COVID-19 pandemic on both adults and children suffering from acute appendicitis in the USA, by Kariya et al. in a review paper analyzing several thousands of cases of appendicitis recorded worldwide, and by Lu et al. in a paper analyzing the effect of COVID-19 pandemic on the progression of appendicitis in adult patients from Wuxi (Jiangsu Province, China) [25,26,27]. However, Köhler et al. reported higher rates of complicated appendicitis in adults treated during the COVID-19 pandemic but did not observe this phenomenon in the case of children [28]. Lastly, differently from many earlier studies that reported that more advanced forms of appendicitis are diagnosed during the pandemic period, Nawacki found no differences in histopathological examination results between pre-pandemic and pandemic groups of adult patients investigated in Kielce (Poland) [29]. 

It should be mentioned that, in general, the risk of perforation is higher in children than in adults, and, as reported by Narsule et al. and Meltzer et al., each hour of delay is associated with a 2% increase in the odds of perforation [30,31]. However, our study did not confirm that the COVID-19 pandemic worsened outcomes for pediatric patients with appendicitis. Such a conclusion is supported by the following facts: the number of complicated cases of appendicitis, the results of basic laboratory tests checking the advancement of inflammatory reactions, and the total duration of hospitalization of patients with appendicitis did not show significant differences between the pre-pandemic period and the pandemic one. Similar findings were also reported by La Pergola et al. in Italy, Gaitero Tristan et al. in Madrid (Spain), Vansevičienė et al. in Kaunas (Lithuania), and the authors of two studies analyzing the patients from the USA, Li and Saleh and Head et al. [22,32,33,34,35]. All these authors found no worsening of outcomes of pediatric appendicitis during the pandemic. Most recently, the same conclusion was also reached by Miscia et al., who stated that the COVID-19 pandemic influenced the time of diagnosis of acute appendicitis in children, severity of inflammation, and type of surgery but did not influence the number of post-operative complications, which implies that the patients were correctly managed [24]. A similar conclusion was also presented by Köhler et al. and Wolf et al. in studies devoted to patients with appendicitis (including adult ones) treated in Germany [36,37]. However, Sartori et al. observed a significant increase in the number of post-operative complications in adult patients treated in Italy at the beginning of the COVID-19 pandemic compared to the pre-pandemic year [38].

### 4.4. The Effects of the COVID-19 Pandemic on the Number of Hospitalizations for Pediatric Conditions

Our study also demonstrated that the outbreak of the COVID-19 pandemic was responsible for a reduction in the number of hospitalizations in the Department of Pediatric Surgery of the Marciniak Hospital in Wrocław (Poland). As reported by Sano et al. (2021), the same tendency was observed in Japan already in the first half of 2020 [39]. Their study demonstrated that the number of hospitalizations for most pediatric conditions decreased because of the COVID-19 outbreak. Only in the case of a single medical condition—acute appendicitis—did the number of hospitalizations remain similar during the pandemic and the pre-pandemic period [39].

These results bear a striking similarity to the results of our present study. In our study, the number of hospitalizations of pediatric patients complaining of abdominal pain also decreased, but the percentage of patients with suspected appendicitis remained nearly the same as before the pandemic. The decrease in the number of patients that were admitted to our Department for observation that took place during the pandemic period was related to more careful qualifications for hospitalization employed during the pandemic time. The criteria for hospitalization were more restricted and based above all on the results of abdomen palpation and ultrasonography. Patients without peritoneal signs or ultrasonography-confirmed appendicitis were discharged with instruction of close monitoring and control after 24 h. As demonstrated by the results of our present study, these modifications of our procedure were not followed by an increase in the severity of appendicitis cases treated during the pandemic. 

### 4.5. Main Strengths and Limitations of Our Study and Perspectives of Future Research

One of the main strengths of our study is its clear focus on a specific population of pediatric patients with appendicitis, a disease which has a well-documented natural progression. This makes meaningful comparisons of disease outcomes across different time periods possible. Additionally, our comparison between the pre-pandemic and pandemic periods provides valuable insights into how the healthcare system’s adaptations implemented during the COVID-19 crisis affected treatment outcomes.

However, our study also has several limitations. First, it was conducted in a single hospital, limiting the generalizability of its results to other regions with different healthcare infrastructures and pandemic responses. Second, the lack of long-term follow-up data means that potential delayed complications in patients treated during the pandemic may not have been captured. Lastly, the socioeconomic and psychological impacts of the pandemic on patients and their caregivers, which could have influenced treatment-seeking behavior, were not fully explored in this study.

We also did not investigate the effects of COVID-19 infection and COVID-19 vaccinations on the triggering of appendicitis in adults and children and the management of acute appendicitis co-occurring with COVID-19 infection, the phenomena reported and discussed, among others, by Georgakopoulou et al., Nawacki, Kaselas et al., and Pantalos et al. [10,11,17,29,40]. However, we would like to point out that the data reported in this study were collected before the start of the distribution of COVID-19 vaccines for children in Poland.

All these issues may give rise to further studies that will broaden our knowledge on numerous important questions, including the impact of COVID-19 and other viral infections on the progression and treatment of other diseases, and the importance of specific legal regulations implemented in medical care on the outcomes of various medical problems.

## 5. Conclusions

This study aimed to assess the impact of the COVID-19 pandemic on pediatric patients with acute appendicitis. Contrary to our initial hypothesis, the outcomes for children with acute appendicitis were not significantly worse during the pandemic period than during the pre-pandemic one. We found no major significant differences in the degree of advancement of appendicitis, inflammatory markers, or overall hospitalization outcomes between these two periods. Despite the expectation that delays caused by the pandemic would result in more severe cases of appendicitis, our data suggest that the stricter admission criteria and improved triage protocols implemented during the pandemic did not negatively affect patient outcomes.

The key conclusion that may be drawn from our study is that the stricter triage criteria introduced during the pandemic helped to reduce unnecessary hospital admissions without negatively impacting patient outcomes. This highlights the potential benefit of implementing such criteria in everyday clinical practice to prevent over-admission and optimize hospital resources.

## Data Availability

The datasets used and/or analyzed during the current study are available from the corresponding author on reasonable request.
